# Genome-Wide Analysis of Exertional Rhabdomyolysis in Sickle Cell Trait Positive African Americans

**DOI:** 10.3390/genes15040408

**Published:** 2024-03-26

**Authors:** Mingqiang Ren, Nyamkhishig Sambuughin, Ognoon Mungunshukh, Daniel Baxter Edgeworth, Daniel Hupalo, Xijun Zhang, Matthew D. Wilkerson, Clifton L. Dalgard, Francis G. O’Connor, Patricia A. Deuster

**Affiliations:** 1 Consortium for Health and Military Performance, Department of Military and Emergency Medicine, F. Edward Hébert School of Medicine, Uniformed Services University, Bethesda, MD 20814, USAdaniel.edgeworth.ctr@usuhs.edu (D.B.E.); francis.oconnor@usuhs.edu (F.G.O.);; 2 Henry M Jackson Foundation for the Advancement of Military Medicine, Inc., Bethesda, MD 20817, USA; 3 Department of Anatomy, Physiology, and Genetics, Center for Military Precision Health, Uniformed Services University, Bethesda, MD 20814, USA

**Keywords:** sickle cell trait, exertional rhabdomyolysis, genome-wide association study, SCL44A3 gene, whole-genome sequencing

## Abstract

Sickle cell trait (SCT), although generally a benign carrier state of hemoglobin S (HbAS), is a risk factor for exertional rhabdomyolysis (ERM), a rare but potentially fatal consequence of highly intense physical exercise, particularly among active-duty military personnel and high-performance athletes. The association between SCT and ERM is poorly understood. The objective of this study was to elucidate the genetic basis of ERM in an SCT-positive African American cohort. SCT-positive African Americans with a personal history of ERM (cases, n = 30) and without history of ERM (controls, n = 53) were enrolled in this study. Whole-genome sequencing was performed on DNA samples isolated from peripheral white blood cells. Participants’ demographic, behavioral, and medical history information was obtained. An additional 131 controls were extracted from SCT-positive subjects of African descent from the 1000 Genomes Project. SCT carriers with ERM were characterized by myotoxicity features, significant muscle involvement dominated by muscle weakness, and severe pain and substantial increase in serum creatine kinase, with a mean value of 50,480 U/L. A distinctive feature of the SCT individuals with ERM was exertional collapse, which was reported in 53.3% of the cases in the study cohort. An important factor for the development of ERM was the duration and frequency of strenuous physical activity in the cases compared to the controls. Whole-genome sequencing identified 79,696 protein-coding variants. Genome-wide association analysis revealed that the p.C477R, rs115958260 variant in the SLC44A3 gene was significantly associated with ERM event in SCT-positive African Americans. The study results suggest that a combination of vigorous exercise and a genetic predisposing factor is involved in ERM.

## 1. Introduction

Approximately 300 million people worldwide [1] and nearly 7.8% of African Americans in the US [2] have sickle cell trait (SCT), a carrier state of sickle hemoglobin S (HbS) that, in HbSS form, causes Sickle Cell Disease (SCD) [3]. Although SCT carrier status is largely considered benign, individuals with SCT are at increased risk for negative health outcomes during intense physical exertion, especially in hot climates and high-elevation locations, or when dehydrated [4]. Our previous epidemiological study with a large cohort (n = 47,944) of active-duty African Americans showed that SCT is associated with a significantly higher risk of exertional rhabdomyolysis (ERM) [2], and this conclusion is also supported by a recent study from the UK Biobank [5]. In addition, cohort studies have demonstrated that military recruits and college athletes with SCT have increased risk of exercise-related death compared to peers without SCT [6,7]. In 2019, a consensus conference we had the opportunity to lead, explored this increased risk of death in those with SCT, outlined critical research gaps [8].

Rhabdomyolysis is an acute and potentially fatal clinical syndrome, which can lead to a variety of complications, including acute kidney injury [9,10]. There are a number of potential etiologies, including exertion, extreme temperature changes, ischemia, infections, immobility, drugs, toxins, endocrine causes, autoimmune reactions, trauma, or genetic conditions [11,12,13]. Inherited muscle disorders associated with rhabdomyolysis are heterogeneous and rare. Genetic variants associated with rhabdomyolysis have been reviewed by Scalco et al. [14]. However, these or other variants are still insufficient to predict an individual’s clinical susceptibility to rhabdomyolysis. Currently, there are limited genomic studies on rhabdomyolysis [12,15,16]. The underlying causes of rhabdomyolysis remain unknown in many cases [12,16,17]. This suggests that additional studies are needed to identify causative disease genes and variants associated with rhabdomyolysis.

ERM is a serious consequence of intense, repetitive, and prolonged physical exercise, particularly among active-duty military personnel and high-performance athletes [18,19]. From 2019 to 2020, the national estimate of hospital visits in the US for ERM was 40,654, which translates to 0.66 visits per 100,000 population. The highest risk groups for ERM are young males and African Americans [20]. Clinically, ERM is most commonly defined as severe myalgia, muscle weakness, and swelling, with sudden elevation of serum creatine kinase (CK) levels with or without the presence of myoglobinuria upon significant exertion [12]. Unaccustomed exercise or exercise in combination with other individual and environmental factors such as dehydration, drugs and toxins, infections, and hyperthermia is a frequent cause of ERM. On the other hand, ERM, especially recurrent ERM, can also be a presentation of inherited metabolic myopathies, intramuscular calcium release, mitochondrial cytopathy, and muscular dystrophies [12,21,22]. However, these rare inherited muscle disorders are not likely a shared cause of ERM associated with SCT. The underlying cause of the variable degree of susceptibility to ERM in SCT individuals remains unknown.

In this study, we aimed to characterize shared clinical features of a series of SCT/ERM cases and to determine genetic markers contributing to ERM in SCT individuals by using a case-control study with whole-genome sequencing (WGS) and exploring genome-wide association approach. To increase the statistical power of the genetic association, we took advantage of SCT-positive genomes from the 1000 Genomes Project data that are widely used in genome-wide association studies (GWAS) [23,24]. We reasoned that the exertion level of our enrolled controls would be comparable to that of SCT individuals from the 1000 Genomes Project, which were added to the control group in our GWAS analysis. We found that a non-synonymous variant leading to c.T1429C:p.C477R (rs115958260) in the SLC44A3 gene was significantly associated with ERM occurrence in SCT-positive subjects. Our study suggests that abnormal function of choline-like transporter 3 (solute carrier transporters) could potentially be associated with ERM development.

## 2. Materials and Methods

### 2.1. Study Participants and Clinical Information

The study protocol was approved by the Uniformed Services University (USU) Institutional Review Board. An informed consent document was obtained from each participant prior to enrollment. We enrolled African American carriers of SCT with a history of ERM (case) and with no history of ERM (control). The study participants were recruited nationwide via referral from military and civilian health care providers. The participants were also invited through advertisements at various treatment facilities, social media, and verbal communications. Following enrollment, participant demographics (age and gender), physical characteristics (height and weight), and behavioral patterns (physical activity, drug and supplement usage), as well as medical and family histories, were obtained through self-reported questionnaires and medical records when available. All cases were evaluated by study physicians in person or via phone interview to obtain clinical history and test results during or after rhabdomyolysis episodes. The enrollment period for this study is from 14 December 2016 to 21 September 2021.

### 2.2. DNA Sample Handling and Whole-Genome Sequencing

Genomic DNA was isolated from peripheral white blood cells by using the QIAamp DNA blood mini kit (Qiagen) following the manufacturer’s instructions. Genomic DNA quantitation was performed using a Qubit^®^ dsDNA HS Assay Kit (Molecular Probes) and measured by an Invitrogen™ Qubit™ 4 Fluorometer. Carrier status of SCT was first validated by genotyping the E7V mutation in the HBB gene by restriction enzyme DdeI. Genotyping of HBB was finally confirmed by Sanger DNA sequencing. DNA library preparation and whole-genome sequencing (WGS) were described per Soltis et al. [25]. WGS was performed on an Illumina HiSeq X System with 151 + 7 + 151 cycle parameters by using HiSeq X HD SBS Kit reagents (Illumina, San Diego, CA, USA). 

### 2.3. Germline Variant Calling

Raw sequencing reads were first converted to standard FASTQ format by using bcl2fastq2 conversion software v2.20 (Illumina). Quality control of the raw reads was performed with FastQC. The GATK package was used for variant calling. The GATK pipeline was based on the best practices workflow for germlines established by the Broad Institute [26]. Briefly, reads were aligned to the reference genome (NCBI GRCh38) using BWA-MEM. Picard tools were then used to sort and mark PCR duplicate reads and generate BAM files. GATK version 4.2.0 was used to recalibrate BAM files with BaseRecalibrator and ApplyBQSR, and to generate VCF and GVCF files with HaplotypeCaller. The VCF files were then used for annotation and analysis. 

### 2.4. Variant Annotation and Filtering

Variant annotation was performed by the Ensembl VEP [27]. Variants were then filtered with various criteria: (1) single nucleotide polymorphism (SNP) call rate  ≥ 95%, (2) a minor allele frequency (MAF) > 1% (estimated using combined datasets from the 1000 Genomes Project and dbSNP154), (3) Hardy-Weinberg equilibrium *p*-value ≥ 1 × 10^−4^, (4) non-synonymous and splice variants, and (5) protein-coding genes (excluded noncoding genes). Variants located in introns, 3′ or 5′ untranslated regions (UTRs), upstream, downstream, or noncoding regions, or those that would cause synonymous mutations and in-frame indels, were mostly not included as the majority of variations occurring in these regions have insufficient evidence. In addition, samples were checked for relatedness and sex mismatch. The relationship was estimated through identity-by-descent (IBD) segments detection with Plink (v1.9) software [28] and further confirmed by using KING (v2.3.0) and PRIMUS (v1.9.0) software [29,30]. Three related subjects with pi-hat threshold > 0.2 were removed from the control group [31].

### 2.5. Case-Control Genome-Wide Association Analysis

PLINK (v1.9) software [28] was used to perform the basic genome-wide association study with sex as a covariate. Additional controls (n = 131) of African descent were extracted from the 1000 Genomes Project, and their genomic variant data were merged with our cohort data for a final GWAS. In total, 106 females and 101 males, in the age range 18–75 years, were included in this study. Considering our small sample size, we intentionally set the significant threshold *p*-values at 5 × 10^−8^, which is more strenuous than Bonferroni correction *p*-value (0.05/79,696 = 6.27 × 10^−7^). The qqman R package was used for graphical representation of the GWAS single-locus analysis results (Manhattan plot). Genetic variants were annotated using the Variant Effect Predictor tool (version 104) [27].

### 2.6. Gene-Based Association Study and Enrichment Analyses

MAGMA software (version 1.10) [32] was used to calculate gene-wise statistics. This software detects multi-marker effects, taking into account the physical distance and linkage disequilibrium between SNPs [32]. These analyses used a 50 kb upstream and downstream window around each gene to capture potential regulatory variants of these genes. Bonferroni correction of *p*-values was performed on the resultant genes (15,694 genes). Finally, 3.2 × 10^−6^ (0.05/15,694) was the *p*-value threshold established for this study for statistical significance. 

## 3. Results 

### 3.1. Study Participant Characteristics

The demographic and behavioral characteristics of the SCT individuals who participated in this study are presented in Table 1. Male subjects comprised 86.7% of the cases, whereas 30.4% of the controls were male. The median age was also significantly different between cases and controls. Cases also showed significantly more strenuous physical activity compared to controls, with cases tending to perform activities frequently and for longer periods of time. These differences between the cases and controls were likely due to the fact that most of the cases (70%) were active-duty Service Members. Cases had lower BMI compared to controls, although the differences were not significant. There were no differences in medical and family histories between cases and controls. All the participants denied frequent use of drugs associated with rhabdomyolysis, such as statin, opioids, and antipsychotics.

Clinical characteristics of the ERM cases are shown in Table 2. Clinical data included peak creatine kinase (CK) levels of greater than 5 times the normal upper limit (200 U/L), presenting symptoms during or after rhabdomyolysis episodes, and other co-morbidities. The mean peak CK was 50,480 U/L, with large variability in range (989–400,000 U/L) among cases. Severe muscle pain was the most common complaint during an episode, followed by weakness/fatigue. Almost half (53.3%) of the cases also reported exertional collapse. Other complications were compartment syndrome, acute kidney injury, and exertional heat illness. Recurrent episodes were observed in 39% of the cases, and the remaining cases were subjects with single episodes.

### 3.2. Single Nucleotide Polymorphisms (SNP) Based on GWAS of ERM in Subjects with SCT

**Figure 1:**
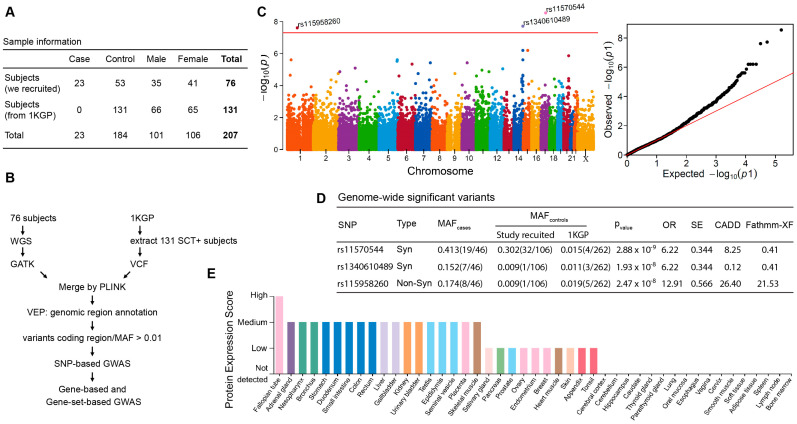
Summary of genome-wide association study of exertion-related rhabdomyolysis in subjects with sickle cell trait (SCT). (**A**) Sample information from enrolled subjects and African-ancestry SCT-positive subjects extracted from the 1000 Genomes Project (1KGP). (**B**) Workflow of data analysis. (**C**) SNP-based Manhattan plot (left, *p* < 5 × 10^-8^) and Q–Q plot (right). The *y*-axis of the former shows the –log10 *p*-values for SNP in the GWAS. The horizontal red line represents the threshold of genome-wide significance (*p* = 5 × 10^-8^). (**D**) The minor allele frequency (MAF) of variants in the case and control groups (number of effect alleles vs. reference alleles in parentheses). Syn:synonymous; Non-Syn: nonsynonymous; CADD: Combined Annotation Dependent Depletion Score (phred); Fathmm-XF: Functional Analysis Through Hidden Markov Models with an eXtended Feature set. (**E**) Tissue-specific patterns of protein expression of SLC44A3 from the Human Protein Atlas project [35].

We performed WGS in 23 of 30 cases and 53 controls (3 of 56 family-related subjects were removed in the final analysis). Variant calling was performed using the GATK best-practices workflow (GATK V4.2.0). We first combined our genomic variant data with subset data of SCT carriers (n = 131) extracted from the 1000 Genomes Project and then performed a genome-wide association study (Figure 1). Because the downstream effect of coding region variants is largely considered more deleterious, we focused only on protein-coding variants in the analysis. A total of 79,696 protein-coding SNPs, which are shared among all samples, were tested for association with ERM, and the results are summarized in Figure 1. The non-synonymous variant of rs115958260 (located in the SLC44A3 gene, exon13:c.T1429C:p.C477R) and both synonymous variants of rs11570544 (located in the CDC27 gene) and rs1340610489 (located in AHNAK2) were associated with ERM at the genome-wide significance threshold (*p* < 5 × 10^−8^) (Figure 1C). These two synonymous variants were removed from further analysis based on our variant filter criteria (Section 2) and low functional scores of CADD (Combined Annotation Dependent Depletion) [33] and Fathmm-XF (Functional Analysis Through Hidden Markov Models with an eXtended Feature set) [34] (Figure 1D).

Detailed analyses of minor allele frequencies (MAF) of rs115958260 demonstrated at least tenfold difference between the cases and controls, both from the enrolled subjects and from presumably healthy SCT individuals from the 1000 Genomes Project (Figure 1D). The overall MAF of this variant in the African-ancestry population is also low (1–2%) in both the 1000 Genomes and gnomAD databases (Supplementary Figure). Functional annotation with in silico scores showed both high CADD and Fathmm-XF integrative scores for rs115958260: 26.4 and 21.53, respectively (Figure 1D and Supplementary Figure), which suggests potentially deleterious effects of the variant. We further screened the remaining 7 SCT/ERM cases, for which whole-genome sequencing was not performed, and found that one case also carried rs115958260. Next, we searched for tissue-specific expression of SLC44A3 protein by exploring data from the Human Protein Atlas project [35]. As illustrated in Figure 1E, high protein expression scores in both skeletal muscle and kidney tissues, which are frequently damaged in ERM, suggests that the SLC44A3 may play an important role in transport of inorganic cations/anions (choline-like transporter) during exercise and muscle contraction.

### 3.3. Gene-Based and Gene-Set-Based GWAS Analyses Show SLC44A3 Significantly Associated with ERM in SCT Carriers

**Figure 2:**
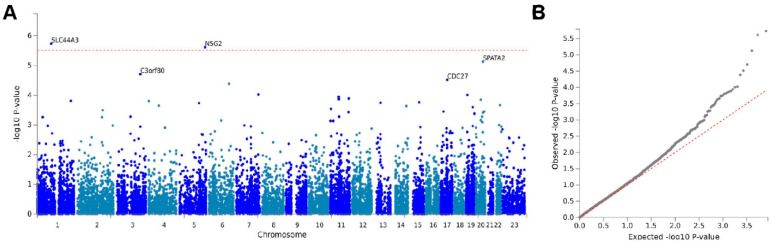
Gene-based genome-wide association analysis. (**A**) Manhattan plot of the gene-based genome-wide association analysis by using a multiple linear principal components regression model (MAGMA). Input SNPs were mapped to 16,187 protein-coding genes. Genome-wide significance (red dashed line) was defined at *p* = 0.05/16187 = 3.089 × 10^−6^. (**B**) Gene-based Q–Q plot.

Next, we performed gene-based genome-wide association analysis by using a multiple linear principal components regression model (MAGMA) [32]. MAGMA analysis demonstrated again that SLC44A3 is significantly (*p* = 1.84 × 10^−6^) associated with ERM, but not for CDC27 (the Bonferroni correction *p*-value = 3.089 × 10^−6^; Figure 2). We further performed gene-set-based genome-wide analysis by using the Reactome pathway database [36]. Solute carrier (SLC)-mediated membrane transport was among the top 5 of 15 pathways (Table 3), which suggests that abnormal function of small molecular transport may serve a role in the development of ERM.

## 4. Discussion

Although largely considered a benign carrier state, SCT is of particular interest to all military and civilian providers due to its association with exertion-related injuries such as ERM, exertional collapse associated with SCT, and sudden death [37,38,39,40]. Fundamental questions addressing etiology, as well as the potential variable risk to various health co-morbidities, such as exertional injuries among those with SCT, remain unanswered. An increasing number of studies, including this one, have shown that certain genetic risk factors contribute to an individual’s variability in the development of ERM [21,41]. However, no studies have been conducted to determine genetic risk factors other than SCT that might predict which SCT carriers are at greatest risk for developing ERM. In the current study, we used whole-genome sequencing to perform GWAS to uncover any genetic factors linked to this association. Both SNP- and gene-based GWAS analyses revealed significant association of the SLC44A3 gene variant rs115958260 with ERM in the SCT-positive African American population. We also characterized shared clinical features of ERM in the series of SCT individuals. SCT carriers with ERM presented with features of myotoxicity symptoms of significant muscle involvement dominated by muscle weakness and severe pain and substantial increase in serum CK, with a mean value of 50,480 U/L. A distinctive feature of SCT individuals with ERM was exertional collapse, which was reported in 53.3% of the cases presented in this study. An important factor for the development of ERM was duration and frequency of strenuous physical activity in cases compared to controls, which suggests that a combination of vigorous exercise and a genetic predisposing factor is involved in ERM. 

The SLC44A3 gene is one of the SLC44 family members (SLC44A1-5), also called the choline-like transporter family [42]. Choline is important for the formation of membrane phospholipids and the neurotransmitter acetylcholine [43]. A choline deficiency caused by mutations in the SCL44A1 gene, a close homolog of SLC44A3, is known to cause neurodegenerative disease, with abnormal regulation of the genes involved in mitochondrial fatty acid transport and β-oxidation, and evidence of disturbances in fatty acid homeostasis [43]. Likewise, a dietary choline deficiency has been associated with muscle damage [44,45]. Further larger-scale genetic association between ERM and SCT, as well as the functional role of the SLC44A3 gene in the development of ERM, will be critically important. 

ERM is a complex syndrome with multiple environmental (unaccustomed exercise, fasting, and hypokalemia) and genetic contributing factors. Abnormalities in the genes encoding proteins in the glycolytic and fatty acid oxidation pathways, calcium dysregulation, and mitochondrial respiratory chains are thought to be involved in the pathogenesis of ERM [12,14,19]. No mitochondrial DNA analysis was performed in this study. However, these inherited disorders are rare and likely not responsible for most cases of ERM. Also, our previous study showed the same hazard ratio as being a SCT carrier. However, the percentage of participants in the present study with smoking history (as shown in Table 1) is small, with no difference between the case and control groups. It is unlikely that smoking status impacted ERM development in this study. Common genetic variants in angiotensin 1 converting enzyme (ACE1), muscle isoform of creatine kinase (CKM), and fast-fiber specific α-actinin 3 (ACTN3) genes have been associated with ERM or muscle symptoms related to ERM. However, these proposed variants of ERM-related genes were not confirmed by this study, similar to the results of other association studies [46,47]. Moreover, it is worth noting that glucose-6-phosphate dehydrogenase (G6PD) deficiency is prevalent among male African Americans, ranging from 10% to 14% [48]. G6PD deficiency in concert with SCT likely precipitated rhabdomyolysis, albeit very rarely [49]. Our current study did not reveal any pathogenic mutation associated with G6PD deficiency or accumulation of deleterious G6PD variants. As a result, our gene-level association analysis found no significant (*p* = 0.68) association between G6PD and ERM.

The percentage of HbS, which ranges from 25% to 45% [50], may affect the hematological and clinical characteristics of SCT, including ERM [51]. However, the Kennedy et al. study suggested that HbS levels in SCT were very unlikely to have any clinical significance [52]. Upon reviewing the literature, we noticed that the majority of studies compared SCT carriers with healthy controls (SCT-negative) without addressing the variability in HbS percentage among SCT individuals [51]. Nevertheless, we cannot exclude the effect of HbS percentage on ERM development without examining it in our study. Additionally, the influence of α-thalassemia or α-globin gene triplication on ERM cannot be excluded since these mutations have been shown to affect HbS concentration in the African American population [53]. 

The association between ERM and SCT has mostly been described in clinical case studies and or epidemiological studies of SCT-associated co-morbidities. To date, there are no GWAS studies on ERM. Clinically significant ERM is rare, and collection of large study cases remains challenging. Another challenge is the phenotypic ascertainment of individuals. The clinical presentation in most cases is nonspecific, but usually includes elevated CK, dark urine secondary to myoglobinuria, and various other health complications. To date, most of the available data regarding the genetic basis of ERM are based on individual gene-specific case reports, which makes it difficult to conclude whether the variants identified are pathogenic. 

Finally, there were no GWAS summary statistics results of ERM/RM in the public databases, such as the NIH GWAS catalog and the UK BioBank. These challenges are also relevant to the limitations of the current study, where sample size was not sufficiently powered for genome-wide variants-based GWAS. This led us to focus only on protein-coding region variants, although filtration of other types of variants certainly impacted the gene-based GWAS. This association study does not confirm the genomic variants previously identified as risk factors for susceptibility to ERM. However, the study design could not provide complete confidence in the control groups not including some individuals with a propensity for ERM or simply having never been challenged with extreme exertion. As a result, there is a possible bias toward “false negatives” in the control groups, and our study could not completely refute those other genomic variants. In addition, our study cannot exclude that rare variants (MAF < 1%) might contribute to ERM development due to the limited sample size. Nevertheless, we clinically characterized African American SCT individuals with ERM and used an unbiased WGS approach to gain a deeper understanding of the association between SCT and exertion-related injuries. 

## 5. Conclusions

The study identified a rare, non-synonymous rs115958260 variant in the SLC44A3 gene, which is significantly associated with ERM in SCT-positive subjects of African American descent. Our study suggests that a combination of vigorous exercise and a genetic predisposing factor is involved in ERM.

## Figures and Tables

**Table 1:** General characteristics of SCT-positive subjects enrolled in the study.

Characteristics	Cases (n = 30)	Controls (n = 56)	*p*-Value
Gender (n, %)			<0.001
Male	26 (86.7)	17 (30.4)	
Female	4 (13.3)	39 (69.6)	
Age at enrollment (years)			
Median (range)	29.5 (18–69)	45 (18–75)	
Mean (SD)	33.8 (14.2)	43.6 (15.7)	0.003
BMI (n, %)			
Mean (SD)	27.2 (6.5)	29.5 (6.6)	0.104
Physical activity %			
Strenuous activity low (≤75%)	45	79	<0.001
Strenuous activity high (≥75%)	55	21	<0.001
Smoking (n, %)	3 (10%)	1 (1.8%)	0.14

**Table 2:** Clinical characteristics of SCT subjects with ERM.

Characteristics	Reported (%)
Creatine kinase (U/L)	
Median (range)	11,000 (989–400,000)
“Coca-Cola”-colored urine	26.7%
Number of episodes	
Single	61%
Recurrent (two or more)	39%
Muscle symptoms during event (n)	
Weakness/fatigue	60%
Pain	88.9%
Pain (mean on a scale of 1–10)	7.4 ± 2.6
Swelling	23.3%
Cramps	30%
Exertional collapse	53.3%
Exertional heat illness	10%
Acute kidney injury	13.3%
Compartment syndrome	16.7%

**Table 3:** Top 15 pathways from gene-set-based genome-wide association analysis using MAGMA with the Reactome database.

	Pathways	Genes	β	SE	*p*-Value
1	SYNTHESIS_OF_PIPS_AT_THE_EARLY_ENDOSOME_MEMBRANE	12	0.923	0.268	0.0003
2	REGULATION_OF_IFNG_SIGNALING	9	0.978	0.308	0.0007
**3**	**SLC-MEDIATED_TRANSMEMBRANE_TRANSPORT**	**200**	**0.216**	**0.069**	**0.0009**
4	SYNTHESIS_OF_PIPS_AT_THE_GOLGI_MEMBRANE	14	0.704	0.228	0.0010
5	INTERFERON_γ_SIGNALING	43	0.438	0.156	0.0025
**6**	**TRANSPORT_OF_VITAMINS,_NUCLEOSIDES,_AND_RELATED_MOLECULES**	**22**	**0.592**	**0.213**	**0.0028**
7	PI_METABOLISM	42	0.381	0.142	0.0036
**8**	**TRANSPORT_OF_NUCLEOTIDE_SUGARS**	**5**	**1.136**	**0.426**	**0.0039**
9	LECTIN_PATHWAY_OF_COMPLEMENT_ACTIVATION	5	1.156	0.444	0.0047
10	GRB2_EVENTS_IN_ERBB2_SIGNALING	16	0.611	0.248	0.0069
11	SYNTHESIS_OF_PIPS_AT_THE_LATE_ENDOSOME_MEMBRANE	9	0.750	0.311	0.0080
**12**	**TRANSPORT_OF_INORGANIC_CATIONS/ANIONS_AND_AMINO_ACIDS/OLIGOPEPTIDES**	**78**	**0.264**	**0.110**	**0.0083**
13	POST_NMDA_RECEPTOR_ACTIVATION_EVENTS	21	0.495	0.207	0.0085
14	KSRP_DESTABILIZES_MRNA	9	0.792	0.339	0.0097
**15**	**TRANSMEMBRANE_TRANSPORT_OF_SMALL_MOLECULES**	**406**	**0.115**	**0.049**	**0.0098**

## Data Availability

All relevant data are within the manuscript and its Supplementary Materials. The data isolated from the 1000 Genomes Project are publicly available from https://www.internationalgenome.org/data, and other genotype data will be available from https://www.ebi.ac.uk/eva (after 30 September 2025) (submission #701876).

## Supplementary Materials

The following supporting information can be downloaded at: https://www.mdpi.com/article/10.3390/genes15040408/s1, Supplementary Figure Functional annotation results of rs115958260 variant.
