# Supplementary material for: Genome-Wide Analysis of Exertional Rhabdomyolysis in Sickle Cell Trait Positive African Americans
Source: Genes (Basel). 2024 Mar 26;15(4):408. doi: 10.3390/genes15040408 (PMC11049803; doi:10.3390/genes15040408)

Supporting information

Supplementary Figure. Functional annotation results of rs115958260 variant. Visualization of the functional annotation of rs115958260 in the histogram using FAVOR online annotation. AF: allele frequency; AFR: African American; AMR: Admixed American; ASJ: Ashkenazi Jewish; EAS: East Asian; EUR: European; FIN: Finnish; NFE: Non-Finnish European; OTH: Other population not assigned; SAS: South Asian.CADD: Combined Annotation Dependent Depletion Score (phred); Fathmm-XF: Functional Analysis Through Hidden Markov Models with an eXtended Feature set.

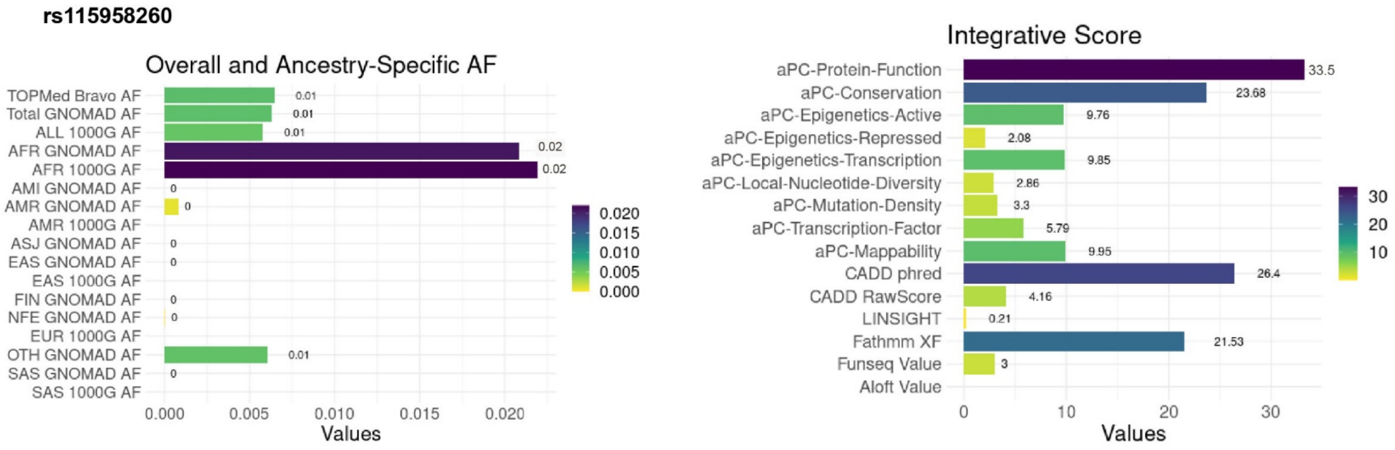

Supplement: Supplementary file 1 [file genes-15-00408-s001.zip › genes-2880699-supplementary.pdf]
